# Secondary prevention implantable cardioverter-defibrillator (ICD) therapy: value in octogenarians

**DOI:** 10.1007/s40520-021-02019-2

**Published:** 2021-11-09

**Authors:** Christian Hauck, Andreas Schober, Alexander Schober, Sabine Fredersdorf-Hahn, Ute Hubauer, Andreas Keyser, Lars Maier, Carsten Jungbauer, Ekrem Ücer

**Affiliations:** 1 Department of Internal Medicine II, University Medical Center Regensburg, 93042 Regensburg, Germany; 2 Department of Cardiothoracic Surgery, University Medical Center Regensburg, 93042 Regensburg, Germany

**Keywords:** Implantable cardioverter defibrillator, Elderly, Octogenarians, Secondary prevention, Mortality

## Abstract

**Background:**

Implantable cardioverter-defibrillator (ICD) therapy is well established for secondary prevention, but studies on the efficacy and safety in elderly patients are still lacking. This retrospective study compared the outcome after ICD implantation between octogenarians and other age groups.

**Methods:**

Data were obtained from a local ICD registry. Patients who received ICD implantation for secondary prevention at our department were included. All-cause mortality, appropriate ICD therapy and acute adverse events requiring surgical intervention were compared between different age groups.

**Results:**

519 patients were enrolled, 34 of whom were aged ≥ 80 years. During the median follow-up of 35 months after ICD implantation 129 patients (annual mortality rate 5.0%) had died, including 16 patients aged ≥ 80 years (annual mortality rate 9.4%). The mortality rate of patients aged ≥ 80 years was significantly higher than that of patients aged ≤ 69 years (*p* < 0.001), but similar to that of patients aged 70–79 years. Age at the time of ICD implantation was an independent predictor of all-cause mortality (*p* < 0.001). 29.7% of patients had appropriate ICD therapy with no difference between age groups. Acute adverse events leading to surgical intervention were low (*n* = 13) and not age-related.

**Conclusion:**

Age is an independent predictor of mortality after ICD implantation for secondary prevention. Mortality rates did not differ significantly between octogenarians and other elderly aged 70–79 years. Appropriate ICD therapy and acute adverse events leading to surgical intervention were not age-related. Implantable cardioverter-defibrillator therapy for secondary prevention seems to be an effective and safe treatment modality in octogenarians.

**Supplementary Information:**

The online version contains supplementary material available at 10.1007/s40520-021-02019-2.

## Introduction

Implantation of an implantable cardioverter-defibrillator (ICD) is the current state-of-the-art therapy to reduce mortality in patients with malign ventricular arrhythmias (VA) as well in survivors of sudden cardiac death due to VA [1–4]. Because of population aging, octogenarians (patient age ≥ 80 years) have become a relevant patient group for an ICD therapy, but this group of patients is still underrepresented in the large ICD trials [1–4]. According to a recent German pacemaker registry report 12.3% of all ICD implantations were performed in octogenarians [5]. Because these patients often suffer from non-cardiac comorbidities leading to non-cardiac death, the benefit of ICD therapy in patients aged ≥ 80 years is still a matter of debate [6].

The current study investigates the clinical outcome of octogenarians after ICD implantation for secondary prevention with regard to all-cause mortality, appropriate ICD therapy and device-related adverse events requiring surgical intervention.

## Methods

### Database

The data of all patients, who received an ICD at our department, were entered into a database. Baseline data include clinical, echocardiographic and device parameters as well as acute adverse events after ICD implantation. The first follow-up visit took place 6 weeks after the implantation. Subsequently, patients had regular follow-up visits every 3–6 months. During each visit a device interrogation was performed by one of our physicians. The device parameters, any episodes of VA, ICD therapies and device-related complications were documented in the registry.

The ICD registry of the University Medical Center Regensburg has been approved by the institutional Ethics Committee and follows the ethical standards laid down in the 1964 Declaration of Helsinki and its later amendments.

### Study group

The current study included 519 patients from the Res-IST Registry who had received an ICD for secondary prevention indication at our department between January 2005 and September 2018. Patients were divided into 6 groups according to their age at the time of ICD implantation [≤ 59 years (*n* = 202), 60–64 years (*n* = 75), 65–69 years (*n* = 81), 70–74 years (*n* = 70), 75–79 years (*n* = 57) and ≥ 80 years (*n* = 34)].

### Outcomes

The primary endpoints of the study were all-cause mortality and appropriate ICD therapy, which was defined as anti-tachycardia pacing (ATP) and/or ICD shock due to ventricular arrhythmias. The safety aspects of ICD implantation were also evaluated, including implantation- and device- related adverse effects such as lead dislocation, lead perforation, device-pocket hematoma, pneumothorax, hemothorax, ICD system infection and pericardial effusion requiring revision surgery during the first month after ICD implantation.

### Statistical analysis

Descriptive data are presented as mean ± standard deviation (SD), medians ± interquartile range (IQR) or percentages. The Mann–Whitney U test was used for continuous variables and the Chi-squared test was used for ordinal variables to test statistical significance. The baseline characteristics of patients aged < 80 years were compared to those of patients aged ≥ 80 years. The selected endpoints were compared between the different age groups (≤ 59 years, 60–64 years, 65–69 years, 70–74 years, 75–79 years and ≥ 80 years) according to Kaplan–Meier analysis and statistical significance was tested with the log-rank test. Furthermore, a Cox-Regression model was used to analyze the influence of age, left ventricular ejection fraction (LVEF), cardiac resynchronization therapy (CRT), ischemic heart disease (IHD), dilated cardiomyopathy (DCM), chronic kidney disease, diabetes mellitus and obesity on all-cause mortality after ICD implantation. A *p* value of ≤ 0.05 was considered statistically significant. For statistical analysis IBM Statistics SPSS Version 25 was used.

## Results

### Baseline characteristics

The baseline characteristics of the 519 patients included in the study are presented in Table 1. Mean age was 61.2 ± 14.5 years and most patients were men (80.3%). The indication for ICD implantation was ventricular fibrillation (VF) in 42.2% and ventricular tachycardia (VT) in 56.3% of patients. 57.3% of patients had ischemic heart disease, and mean LVEF was 38 ± 14%. Patients aged ≥ 80 years had more often ischemic heart disease, permanent atrial fibrillation and chronic kidney disease as well as a lower body mass index. The rate of patients with DCM, other cardiomyopathies or channelopathies and obesity was significantly higher in the age group < 80 years.

**Table 1:** Baseline characteristics (compared age < 80 years and ≥ 80 years; *p* value ≤ 0.05 considered statistically significant)

	All (*n* = 519)	< 80 years (*n* = 485)		≥ 80 years (*n* = 34)	*p* value
Age	61.2 (± 14.5)	59.7 (± 13.8)		82.6 (± 2.2)	< 0.001
Men	417 (80.3%)	390 (80.4%)		27 (79.4%)	0.89
BMI (kg/m^2^)	27.4 (± 5.1)	27.5 (± 5.1)		25.3 (± 3.4)	0.02
LVEF	38% (± 14)	38% (± 14)		37% (± 9)	0.28
ICD indication VF	219 (42.2%)	209 (43.1%)		10 (29.4%)	0.12
ICD indication VT	292 (56.3%)	268 (55.3%)		24 (70.6%)	0.08
ICD indication others^a^	8 (1.5%)	8 (1.6%)		0	0.45
Single-chamber ICD	327 (63.0%)	305 (62.9%)		22 (64.7%)	0.83
Dual-chamber ICD	147 (28.3%)	139 (28.7%)		8 (23.5%)	0.52
S-ICD	13 (2.5%)	13 (2.7%)		0	0.33
CRT	32 (6.2%)	28 (5.8%)		4 (11.8%)	0.16
IHD	297 (57.3%)	272 (56.1%)		25 (73.5%)	0.05
DCM	148 (28.5%)	145 (29.9%)		3 (8.8%)	0.01
Others^b^	95 (18.3%)	93 (19.2%)		2 (5.9%)	0.05
History of MI	198 (38.2%)	183 (37.7%)		15 (44.1%)	0.74
Atrial fibrillation	178 (34.3%)	159 (32.8%)		19 (55.9%)	0.006
Paroxysmal AFib	89 (17.1%)	84 (17.3%)		5 (14.7%)	0.70
Persistent AFib	26 (5.0%)	25 (5.2%)		1 (2.9%)	0.57
Permanent AFib	63 (12.1%)	50 (10.3%)		13 (38.2%)	< 0.001
Diabetes	135 (26.0%)	125 (25.8%)		10 (29.4%)	0.64
Hypertension	323 (62.2%)	298 (61.4%)		25 (73.5%)	0.16
Obesity (BMI ≥ 30)	125 (24.1%)	122 (25.2%)		3 (8.8%)	0.03
Hyperlipidemia	267 (51.4%)	248 (51.1%)		19 (55.9%)	0.59
History of stroke	67 (12.7%)	63 (12.8%)	4 (11.8%)		0.84
PAD	54 (10.4%)	52 (10.7%)	2 (5.9%)		0.37
Carotid stenosis	23 (4.4%)	20 (4.1%)	3 (8.8%)		0.20
CKD	120 (23.1%)	107 (22.1%)	13 (38.2%)		0.03
COPD	42 (8.1%)	38 (7.8%)	4 (11.8%)		0.42
ACE/AT1/ARNI	389 (75.0%)	363 (74.8%)	26 (76.5%)		0.83
Beta blocker	417 (80.3%)	390 (80.4%)	27 (79.4%)		0.89
Spironolactone	247 (47.6%)	234 (48.2%)	13 (38.2%)		0.26
Diuretics	334 (64.4%)	309 (63.7%)	25 (73.5%)		0.25
Amiodarone/Sotalol	43 (8.2%)	39 (7.9%)	4 (11.8%)		0.45
Digitalis	47 (9.1%)	43 (8.9%)	4 (11.8%)		0.57

*BMI* body mass index, *LVEF* left ventricular ejection fraction, *ICD* implantable cardioverter-defibrillator, *VF* ventricular fibrillation, *VT* ventricular tachycardia, *S-ICD* subcutaneous implantable cardioverter-defibrillator, *CRT* cardiac resynchronization therapy, *IHD* ischemic heart disease, *DCM* dilated cardiomyopathy, *MI* myocardial infarction, *AFib* atrial fibrillation, *PAD* peripheral artery disease, *CKD* chronic kidney disease, *COPD* chronic obstructive pulmonary disease

ICD indication others^a^: patients with an out of hospital cardiac arrest (OHCA) and high probability of a primary rhythmogenic cause though first documented rhythm was asystole, pulseless electrical activity (PEA) or sinus rhythm

Others^b^: primary VF: 31 (6.0%), myocarditis: 13 (2.5%), hypertrophic (obstructive) cardiomyopathy (H(O)CM): 11 (2.1%), secondary cardiomyopathy: 12 (2.3%), long-QT-syndrome: 10 (1.9%), arrhythmogenic right ventricular dysplasia (ARVD): 6 (1.2%), Tako Tsubo cardiomyopathy: 6 (1.2%), Brugada syndrome: 3 (0.6%), non-compaction cardiomyopathy: 2 (0.4%), short-QT-syndrome: 1 (0.2%)

### Mortality

**Fig. 1:**
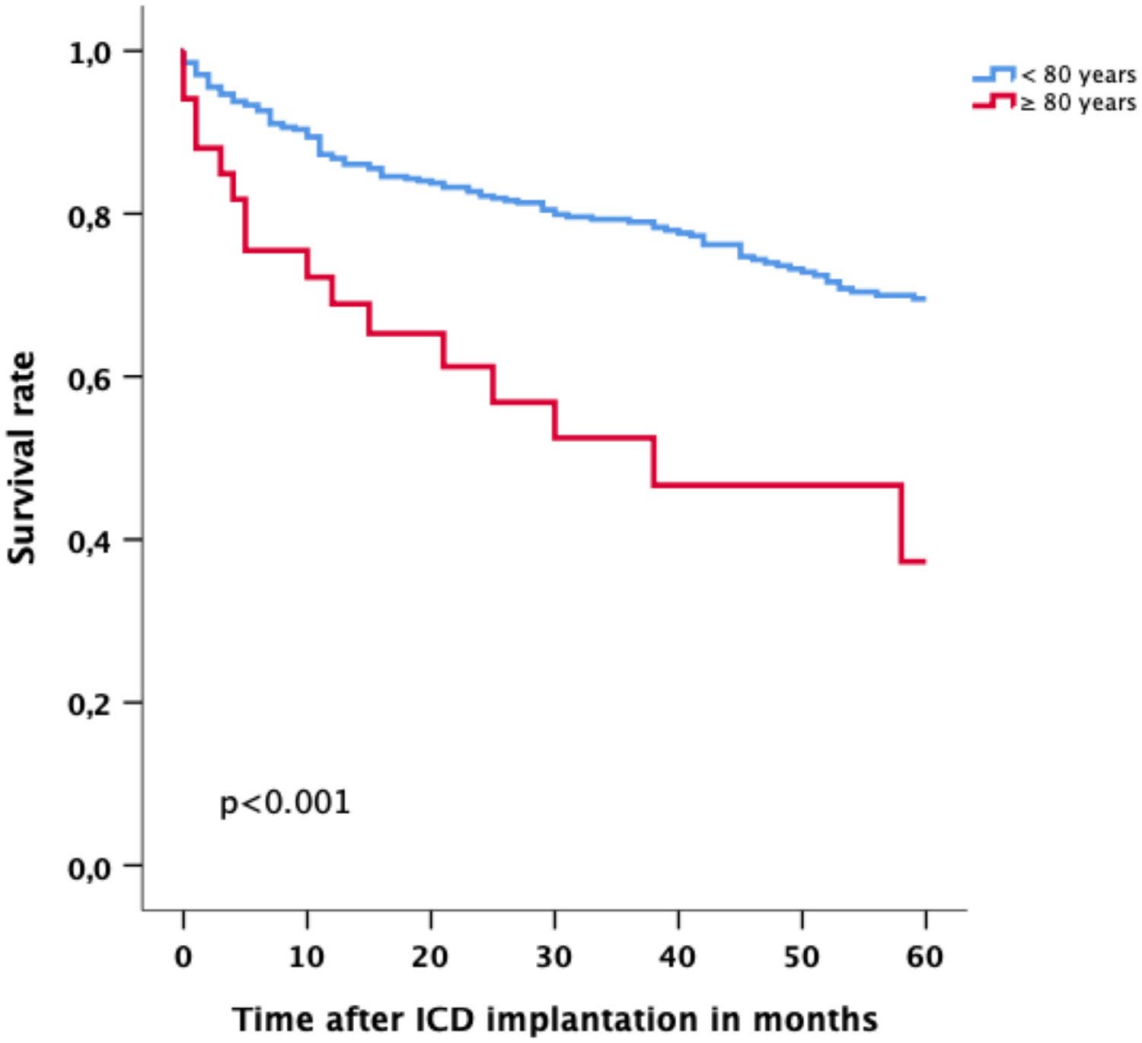
Survival rate of patients with an age ≥ 80 years compared to patients with an age < 80 years

During the follow-up of 35 months (IQR 11–60 months) after ICD implantation 129 (24.9%) patients in the study group had died, 16 of whom were octogenarians. The annual mortality rate was 5.0% for the entire study group, 9.4% for octogenarians, and 4.7% for patients aged < 80 years. According to Kaplan–Meier analysis, the mortality rate in the age group ≥ 80 years was significantly higher than that of patients aged < 80 years (*p* < 0.001) (Fig. 1). Patients were then divided into subgroups according to their age in 5-year steps. A further analysis of the annual mortality rates of these different age groups showed significantly lower annual mortality rates for patients aged < 70 years than for octogenarians [≤ 59 years (2.4%; < 0.001), 60–64 years (3.5%; *p* < 0.001), 65–69 years (4.9%; *p* = 0.002)] but no significant difference between octogenarians and patients aged 70–79 years of age [70–74 years (9.4%; *p* = 0.39); 75–79 years (8.1%; *p* = 0.30)] (Table 2).

**Table 2:** Mortality after ICD implantation (compared to ≥ 80 years; *p* ≤ 0.05 considered statistically significant)

Age groups	Mortality (129/519, 24.9%)	Annual mortality rate (5.0%)	*p* value
< 80 years	113/485 (23.3%)	4.7%	< 0.001
≤ 59 years	24/202 (11.9%)	2.4%	< 0.001
60–64 years	13/75 (17.3%)	3.5%	< 0.001
65–69 years	20/81 (24.7%)	4.9%	0.002
70–74 years	33/70 (47.1%)	9.4%	0.39
75–79 years	23/57 (40.4%)	8.1%	0.30
≥ 80 years	16/34 (47.1%)	9.4%	

### Predictors of all-cause mortality

In a multivariate Cox regression model, age at the time of ICD implantation (*p* < 0.001) as well as chronic kidney disease (*p* = 0.05) were independent predictors of all-cause mortality but not LVEF (*p* = 0.29), diabetes (*p* = 0.44), obesity (*p* = 0.84), CRT therapy (*p* = 0.25), IHD (*p* = 0.72) and DCM (*p* = 0.67) (Table 3).

**Table 3:** Results of univariate and multivariate Cox regression model

	Univariate analysis				Multivariate analysis			
			95% CI				95% CI	
	*p* value	HR	Lower	Upper	*p* value	HR	Lower	Upper
Age	< 0.001	1.05	1.04	1.07	< 0.001	1.05	1.02	1.07
CKD	< 0.001	2.92	2.07	4.14	0.05	1.61	1.01	2.57
Diabetes	0.01	1.67	1.18	2.41	0.44	1.21	0.75	1.93
IHD	0.24	1.24	0.87	1.76	0.72	0.86	0.54	1.37
DCM	0.54	0.89	0.60	1.31	0.67	1.00	0.43	2.34
Obesity	0.15	0.73	0.47	1.12	0.84	0.89	0.52	1.52
LVEF	0.27	0.99	0.97	1.01	0.29	0.99	0.97	1.01
CRT	0.66	1.32	0.67	2.61	0.25	0.52	0.16	1.64

*LVEF* left ventricular ejection fraction, *CRT* cardiac resynchronization therapy, *IHD* ischemic heart disease, *DCM* dilated cardiomyopathy, *CKD* chronic kidney disease

### Appropriate ICD therapy due to ventricular arrhythmias

During the follow-up, 29.7% of all patients had received appropriate ICD therapy because of ventricular arrhythmias (annual rate 5.9%). 19.1% of patients were treated with both ATP and ICD shock or ICD shock alone and 10.6% of patients with ATP without ICD shock.

**Fig. 2:**
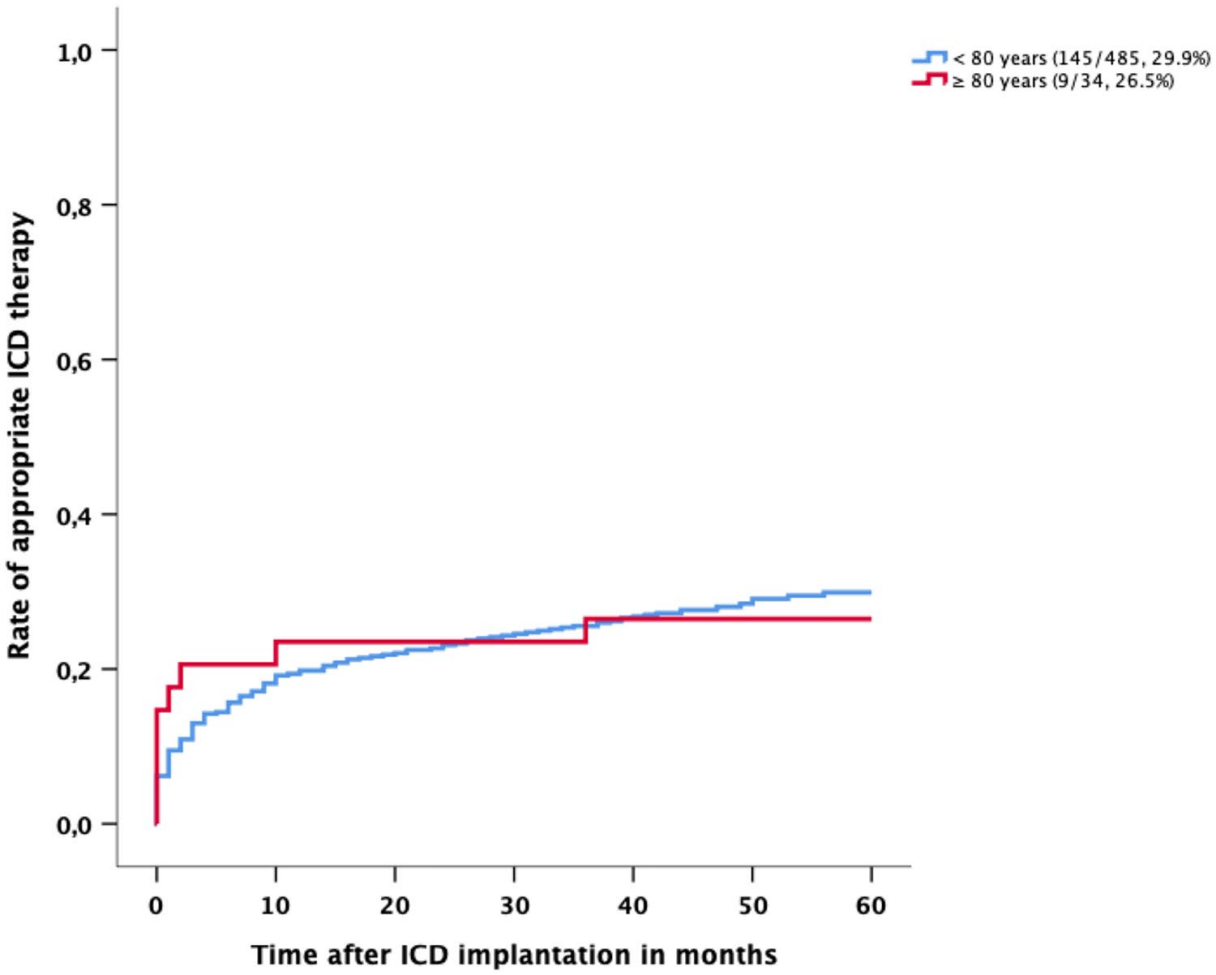
Rate of appropriate ICD therapy after ICD implantation in the age group < 80 years and ≥ 80 years

26.5% of octogenarians received appropriate ICD therapy (annual rate 5.3%). 20.5% had ventricular arrhythmias and were treated with ATP and ICD shock or ICD shock alone and 6.0% with ATP without ICD shock. The rate of ICD therapy did not show any age-related difference between patients aged ≥ 80 years and younger patients (*p* = 0.80) (Fig. 2).

A comparison of the rate of ICD therapy between all age groups did not yield any significant difference between the groups [≤ 59 y (31.2%; *p* = 0.70); 60–64 y (29.3%; *p* = 0.85); 65–69 y (24.7%; *p* = 0.70); 70–74 y (35.7%; *p* = 0.41) and 75–79 y (26.3%; *p* = 0.83)].

### Device-related adverse events requiring surgical intervention

The different age groups did not significantly differ with regard to safety in the first month after ICD implantation. Event rates were very low and included lead dislocation (*n* = 9), ICD system infection (*n* = 2), pocket hematoma (*n* = 2) and hemothorax (*n* = 1). 1 patient developed both lead dislocation and pocket hematoma. None of the patients in the entire study group had developed lead perforation, pneumothorax, or pericardial effusion (Table 4).

**Table 4:** Device-related adverse events requiring surgical intervention (compared to ≥ 80 years; *p* ≤ 0.05 considered statistically significant)

Age groups	Adverse events (*n* = 13/519; 2.5%)	*p* value
≤ 59 years	4/202 (2.0%)	0.72
60–64 years	3/75 (4.0%)	0.79
65–69 years	2/81 (2.5%)	0.89
70–74 years	0/70 (0%)	0.15
75–79 years	3/57 (5.3%)	0.60
≥ 80 years	1/34 (2.9%)	

## Discussion

Our single-center registry study showed a high mortality rate in octogenarians with ICD therapy for secondary prevention. Age at the time of ICD implantation was an independent risk factor for mortality during the subsequent 5 years as was chronic kidney disease. There was no age-related difference in appropriately treated ventricular arrhythmias by ICD therapy. ICD treatment did not result in any higher device-related complications leading to surgical intervention in the octogenarians than in younger patient groups. Therefore, our study showed ICD therapy to be a safe treatment option for patients ≥ 80 years.

### ICD therapy in elderly patients

The effectiveness and safety of ICD therapy for secondary prevention in elderly patients is still not clear because of the underrepresentation of this patient group in the large randomized trials [1–3]. Because of population aging, however, there is a growing need for more information about the effectiveness and safety of ICD therapy in the elderly, especially in octogenarians. Previous studies have shown divergent results regarding the benefit of ICD therapy for secondary prevention in the elderly.

In a population-based registry study in Ontario, Canada, the outcome of ICD therapy for secondary prevention was compared between 159 patients aged ≥ 80 years and patients of other age groups. Over a median follow-up of 670 days after ICD implantation, age was an independent predictor of mortality, but the number of appropriate ICD therapies, especially ICD shocks, did not differ between the different age groups [7]. Those results are very similar to our findings; the main differences to our study are the shorter follow-up duration (670 days) and the larger number of patients. A meta-analysis of three large secondary prevention trials comparing ICD to Amiodarone (AVID [3], CASH [2] and CIDS trial [1]) did not yield any significant benefit of ICD therapy over Amiodarone regarding all-cause mortality and absence of arrhythmic death in patients aged ≥ 75 years [6]. In fact, all-cause mortality in elderly patients in the three above-mentioned studies was mainly caused by death from progressive heart failure during the first year after ICD implantation. The possible benefit of ICD therapy regarding the adequate treatment of life-threatening ventricular arrhythmias, which was not age related in our study, may become more evident over a longer lifespans.

The DANISH trial (Defibrillator Implantation in Patients with Nonischemic Systolic Heart Failure) recently described a beneficial effect of ICD therapy only in patients with nonischemic heart failure aged < 59 years [8]. Despite the fact that the DANISH trial dealt with a completely different patient population (primary prevention patients with dilated cardiomyopathy only) and included a large number of patients with cardiac resynchronization therapy, advanced age was still a predictor of all-cause mortality.

A subgroup analysis of the three large primary prevention trials of ICD therapy (MADIT-II, SCD-Heft and COMPANION) showed a beneficial effect on mortality in older patients. A sub-study of the MADIT-II trial [9] with 204 patients aged ≥ 75 years who had received an ICD for primary prevention showed a non-significant reduction in mortality. Similarly, a subgroup analysis of SCD-Heft [10] and COMPANION [11] showed that patients aged > 65 years may benefit from an ICD therapy. The higher efficacy of ICD in reducing death in the elderly in primary prevention trials may be explained by the lower number of co-morbidities in these patients. Severe comorbidities such as renal failure or other systemic diseases may result in ventricular arrhythmias due to hormonal and electrolyte changes, which, in turn can result in higher mortality. In our study, age at the time of ICD implantation was an independent predictor of mortality. Patients aged > 70 years had a similar mortality rate as octogenarians; thus, co-morbidities should be taken into account when selecting patients aged > 70 years for ICD therapy. The all-cause annual mortality rate of 5.0% of all our patients was lower than the rates in other secondary prevention trials [1–3], which eventually may be explained by medical advances in the treatment of heart failure and coronary heart disease. During the first year after ICD implantation, ten patients in the octogenarian group died resulting in a rather high one-year mortality rate of 29.4%. This shows, that in a real-world clinical setting, it is obviously difficult to assess life expectancy correctly especially in elderly patients. This is an important issue as current guidelines recommend ICD therapy for secondary prevention only for patients, who are expected to survive for more than 1 year with good functional status and quality of life. Patients with serious comorbidities, who are unlikely to survive more than 1 year, should not receive an ICD therapy [12]. Therefore, one must be cautious when evaluating elderly patients for ICD implantation and a screening for comorbidities that are limiting life expectancy to less than 1 year should be made by a multidisciplinary team approach. On the other side, the 1-year mortality rate was also high in patients aged 70–74 years (22.9%) and in patients aged 75–79 years (21.2%). A possible explanation might be, that ventricular arrhythmia, which are the reason for secondary prevention ICD implantation, can be an indicator for a progress of an underlying cardiovascular disease, which can be an important limiting factor for life expectancy in older patients. Besides, the possibility that the ICD can be deactivated should be discussed as a part of the patient’s informed consent for ICD implantation. For example, ICD deactivation in patients with a terminal illness may be ethically permissible to avoid painful ICD shocks [13].

Over 5 years 29.7% of our patients had undergone appropriate ICD therapy. This rate is lower than the rates in other ICD trials [1–3, 7], which may be a result of novel ICD programming strategies with longer detection times and higher cut-off rates [14]. No age-related difference could be seen for appropriate ICD therapy, proving the effectiveness of ICD therapy in treating ventricular arrhythmias in the elderly, which was comparable to other studies [7].

The number of device-related adverse events requiring surgical intervention was low across all age groups, so that the implantation procedure itself does not seem to involve any increased risk in elderly patients.

In our study the use of a single-lead ICD was rather high (63%), while CRT devices were implanted in only 6.2% of our patients. One possible explanation might be, that the mean LVEF in our study group was 38% and the current guidelines recommend CRT for patients with a left bundle branch block and a LVEF ≤ 35% [15]. Moreover, our study included patients who had received an ICD at our department from January 2005. At that time CRT was not as well established as it is today.

If elderly patients are eligible for a CRT, it is important to discuss whether one should implant a CRT-P or a CRT-D. Because the trials, which have investigated this topic, have included only patients with a primary prevention indication [8, 11], no scientific data exist for CRT-P for patients with a secondary prevention indication. It is worth considering, whether the potential rhythm stabilizing effect of resynchronization therapy might be a relevant therapeutic concept in octogenarians. But as scientific data are missing and ICD has been proven to reduce the risk of arrhythmic death, the current guidelines recommend a CRT-D for patients who are eligible for CRT and have a secondary prevention indication for ICD therapy [12, 15].

The strength of the current study compared to already existing scientific data is the long follow-up with a median of 35 months. The large scale of clinical baseline characteristics in our registry enables the identification of confounders that may influence the outcome after ICD implantation in elderly patients. Outcome after ICD implantation may be influenced by significantly more frequent concomitant diseases in patients aged ≥ 80 years. Our study included a real-life cohort; therefore, it is possible that older patients, especially octogenarians, who were eligible for ICD therapy were more carefully selected with regard to concomitant diseases and their general health status than younger patients. This selection indicates that for octogenarians comorbidities, life expectancy, the general health status, and patient preferences need to be individually evaluated before a decision for or against an ICD therapy can be made. However, larger prospective randomized controlled trials are warranted to test the benefit of ICD therapy for secondary prevention in patients aged ≥ 80 years.

## Limitations

Our study group of octogenarians was rather small; in the absence of larger studies, our data may still be useful for evaluating the value of ICD therapy in elderly patients. The retrospective non-randomized study design includes the risk of selection bias because different factors that might have played a role in the decision process for or against an ICD therapy in certain patients cannot be evaluated retrospectively. We included patients over a period of 13 years, during which the advances in heart failure therapy, therapy for IHD, and advancement in ICD programming might have influenced patient outcome. There was also no significant difference in the drug therapy for heart failure, which otherwise might have affected outcome. Furthermore, we had no control group of older patients who did not receive ICD therapy or were treated with antiarrhythmic therapy; for this reason, we do not know if other less invasive strategies may also be effective in elderly patients. Moreover, we cannot provide data about the cause of death because this information was not collected as part of the ICD registry. Finally, this study was a single-center study; thus, we do not know if our results can be generalized for other medical centers, which may have other strategies for selecting patients eligible for ICD therapy or may use other ICD programming to treat ventricular arrhythmias.

## Conclusion

This single-center registry study showed that age at the time of ICD implantation is an independent risk factor for all-cause mortality in patients with ventricular arrhythmia who receive ICD therapy for secondary prevention. On the other hand, the rate of appropriately delivered ICD therapy to treat ventricular arrhythmias and the number of adverse events leading to revision surgery did not show any age-related differences. In summary, ICD therapy for secondary prevention seems to be an effective and safe therapy in elderly patients including octogenarians, who have higher all-cause mortality rates than younger patients due to comorbidities.

## Supplementary Information

Below is the link to the electronic supplementary material.

Supplementary file1 Supplement Figure 1: Survival rate after ICD implantation compared by different age groups (PDF 36 KB)

Supplementary file2 Supplement Figure 2: Rate of appropriate ICD therapy after ICD implantation in all age groups (PDF 43 KB)

## Data Availability

Available from the corresponding author upon request.
