# Supplementary figures and images for: Secondary prevention implantable cardioverter-defibrillator (ICD) therapy: value in octogenarians
Source: Aging Clin Exp Res. 2021 Nov 9;34(5):1073–80. doi: 10.1007/s40520-021-02019-2 (PMC9135875; doi:10.1007/s40520-021-02019-2)

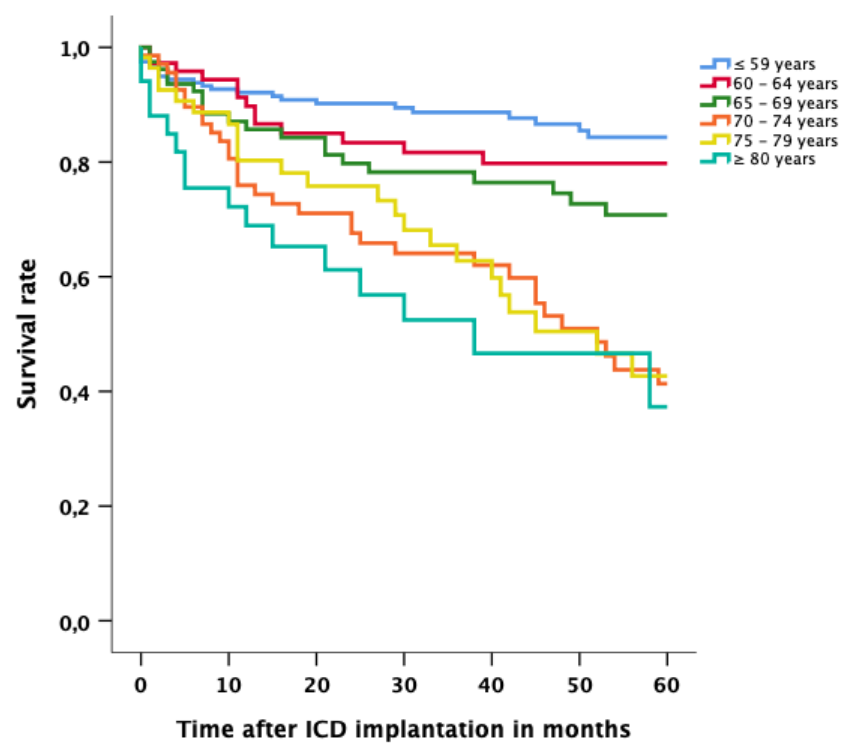

Supplement: Supplementary file 1 — Supplementary file1 Supplement Figure 1: Survival rate after ICD implantation compared by different age groups (PDF 36 KB) [file 40520_2021_2019_MOESM1_ESM.pdf]

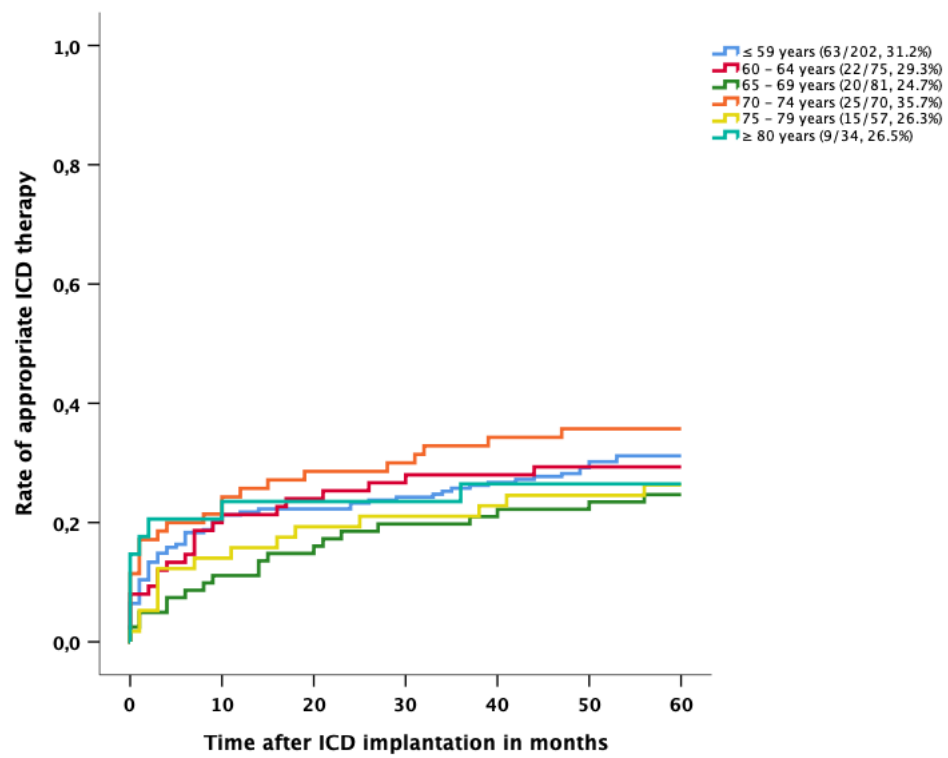

Supplement: Supplementary file 2 — Supplementary file2 Supplement Figure 2: Rate of appropriate ICD therapy after ICD implantation in all age groups (PDF 43 KB) [file 40520_2021_2019_MOESM2_ESM.pdf]
